# Global whole-genome comparison and analysis to classify subpopulations and identify resistance genes in weedy rice relevant for improving crops

**DOI:** 10.3389/fpls.2022.1089445

**Published:** 2023-01-10

**Authors:** Zhenyun Han, Fei Li, Weihua Qiao, Xiaoming Zheng, Yunlian Cheng, Lifang Zhang, Jingfen Huang, Yanyan Wang, Danjing Lou, Meng Xing, Weiya Fan, Yamin Nie, Wenlong Guo, Shizhuang Wang, Ziran Liu, Qingwen Yang

**Affiliations:** ^1^National Key Facility for Crop Gene Resources and Genetic Improvement, Institute of Crop Sciences, Chinese Academy of Agricultural Sciences, Beijing, China; ^2^National Nanfan Research Institute (Sanya), Chinese Academy of Agricultural Sciences, Sanya, China; ^3^International Rice Research Institute, Metro Manila, Philippines

**Keywords:** weedy rice, population classification, structural variation, WRKY, NBS

## Abstract

Common weedy rice plants are important genetic resources for modern breeding programs because they are the closest relatives to rice cultivars and their genomes contain elite genes. Determining the utility and copy numbers of *WRKY* and nucleotide-binding site (*NBS*) resistance-related genes may help to clarify their variation patterns and lead to crop improvements. In this study, the weedy rice line LM8 was examined at the whole-genome level. To identify the *Oryza sativa japonica* subpopulation that LM8 belongs to, the single nucleotide polymorphisms (SNPs) of 180 cultivated and 23 weedy rice varieties were used to construct a phylogenetic tree and a principal component analysis and STRUCTURE analysis were performed. The results indicated that LM8 with admixture components from *japonica* (GJ) and *indica* (XI) belonged to GJ-admixture (GJ-adm), with more than 60% of its genetic background derived from XI-2 (22.98%), GJ-tropical (22.86%), and GJ-subtropical (17.76%). Less than 9% of its genetic background was introgressed from weedy rice. Our results also suggested LM8 may have originated in a subtropical or tropical geographic region. Moreover, the comparisons with Nipponbare (NIP) and Shuhui498 (R498) revealed many specific structure variations (SVs) in the LM8 genome and fewer SVs between LM8 and NIP than between LM8 and R498. Next, 96 *WRKY* and 464 *NBS* genes were identified and mapped on LM8 chromosomes to eliminate redundancies. Three *WRKY* genes (*ORUFILM02g002693*, *ORUFILM05g002725*, and *ORUFILM05g001757*) in group III and one RNL [including the resistance to powdery mildew 8 (RPW8) domain, NBS, and leucine rich repeats (LRRs)] type *NBS* gene (*ORUFILM12g000772*) were detected in LM8. Among the *NBS* genes, the RPW8 domain was detected only in *ORUFILM12g000772*. This gene may improve plant resistance to pathogens as previously reported. Its classification and potential utility imply LM8 should be considered as a germplasm resource relevant for rice breeding programs.

## 1 Introduction

The genus *Oryza* comprises plants with an evolutionary history spanning 15 million years, during which they underwent both natural selection and artificial selection. Weedy rice (*Oryza sativa* f. *spontanea*), which is a conspecific relative of cultivated rice that commonly grows in close proximity to cultivated rice, has certain wild rice characteristics that combine to form diverse features (Li et al., 2017; Wu et al., 2021). Previous research indicated that hybridizations between crops and weeds are relatively uncommon, and alleles for excellent agronomic traits in compatible rice plants may be introgressed into weed populations (Lu et al., 2016; Merotto et al., 2016). According to a recent analysis of genome sequencing data, Chinese weedy rice lines independently de-domesticated from locally cultivated rice varieties and were modified by balancing selection during the de-domestication process (Qiu et al., 2017). Meanwhile, Li et al. (2017) detected two strains of weedy rice in the USA that originated from Asian *indica* and *aus* rice plants *via* de-domestication events. On the basis of population genomic analyses, weedy rice at high latitudes and weedy rice at middle latitudes were grouped with *japonica* and *indica* accessions, respectively (Sun et al., 2019). To date, the commonly used rice reference genomes are from Nipponbare (NIP; Kawahara et al., 2013), 93-11 (International Rice Genome Sequencing Project, 2005), Shuhui498 (R498; Du et al., 2017), as well as Zhenshan 97 and Minghui 63 (Zhang J W et al., 2016). To accelerate the identification of beneficial alleles in weedy rice potentially useful for enhancing cultivated rice, the genomes of WR04-6 (Sun et al., 2019) and LM8 (Li et al., 2021), which are two weedy rice lines, have been assembled.

Unlike animals, which can move toward the most appropriate areas within their habitats and avoid diverse stresses, plants are sessile and continuously exposed to various abiotic and biotic stresses. Accordingly, identifying genetic variations in resistant germplasms and clarifying complex pathways regulating resistance are critical for the breeding of resistant crop species. The immunity network and mechanisms underlying the adaptation of plants to adverse environmental stimuli remain to be thoroughly characterized, although WRKY and NBS genes resistance genes are confirmed contributors. Specifically, WRKY-encoding proteins, also as one of transcription factors (TFs), are involved in regulating plant responses to multiple stresses, including pathogen or bacterial infections, drought, cold, freezing, and wounding (Li et al., 2020). They usually contain a DNA-binding region comprising the highly conserved WRKYGQK sequence (i.e., WRKY domain) at the N-terminus as well as a zinc finger structure (CX_4-5_CX_22-23_HXH) (Eulgem et al., 2000). Both the W-box region with the consensus sequence (C/T)TGAC(T/C) and the sugar-responsive element comprising TAAAGATTACTAATAGGAA in the downstream target gene promoters are specifically recognized by WRKY TFs, resulting in activated transcription (Sun et al., 2003). The WRKY TFs in group I contain two WRKY domains, whereas the group II and III members have one WRKY domain. The WRKY TFs in groups II and III may be distinguished by their C_2_H_2_ and C_2_HC zinc fingers, respectively (Chen et al., 2017). The WRKY domain has been detected in two Arabidopsis TIR-NBS-LRR (TNL) proteins (Narusaka et al., 2009).

In fact, the TIR-NBS-LRR proteins, which are also called TNL proteins, consist of a nucleotide-binding (NB) domain and C-terminal leucine rich repeats (LRRs) along with a Toll/interleukin-1 receptor/resistance (TIR) domain in the N-terminal region (Collier and Moffett, 2009). The presence of a coiled-coil (CC) domain instead of a TIR domain in the NBS-LRR N-terminal region results in a CC-NBS-LRR (CNL) protein (Collier and Moffett, 2009). Apart from TNL and CNL genes, RPW8-NBS-LRR (RNL) represent a special N-terminal domain known as RPW8 (resistance to powdery mildew 8) domain containing NBS-LRR region (Zhang Y M et al., 2016). The NB and LRR domains are separated by an ARC (Apaf-1, R protein, and CED-4) domain (Collier and Moffett, 2009). The functional central nucleotide-binding pocket includes the NB and ARC domains (Collier and Moffett, 2009). During the co-evolution with pathogenic microorganisms, plants evolved a series of resistance (*R*) and avirulence genes that protect against infections. Similar to other crops, rice has evolved two defense strategies, namely pathogen-associated molecular pattern (PAMP)-triggered immunity (PTI) and effector-triggered immunity (ETI) (Dodds and Rathjen, 2010). Of the known *R* genes, *NBS* genes are the most prevalent and they encode proteins with important roles in the ETI system (Meyers et al., 2003). The WRKY TFs and NBS proteins in *Oryza* species have been identified and analyzed, including those in cultivated rice and wild rice; however, those in weedy rice species have not been studied (Ross et al., 2007; Xu et al., 2016; Rawal et al., 2018).

The LM8 weedy rice line was named because its pericarp is green until maturity. There is increasing interest in this weedy rice line among breeders because of its green pericarp and small grains, which may be preferred by consumers. In this study, LM8 was used for a whole-genome analysis after a high-quality genome was assembled. The subpopulation classification of LM8 was completed using cultivated *japonica* and *indica* strains. Moreover, a comparative analysis was performed using NIP and R498 reference genomes. In addition, the weedy rice *WRKY* and *NBS* gene family members were identified and collated for a comprehensive genome-wide analysis and comparison with diverse species. Phylogenetic relationships and the conserved motifs among families were investigated to provide useful insights into the conserved regulator. On the basis of our study findings, LM8 may be used to clarify the rice de-domestication process, while also serving as a resource for plant biotechnologists and breeders interested in enhancing rice traits through genetic modifications.

## 2 Materials and methods

### 2.1 Plant materials and data selection

The 203 *Oryza* species used for the subgroup classification of LM8 included 180 cultivated varieties and 23 weedy rice varieties. The *O. sativa* groups *japonica* and *indica* were designated as GJ and XI, respectively. Using the 3K database (Wang et al., 2018; https://registry.opendata.aws/3kricegenome/), 20 cultivated varieties were randomly selected from the GJ-adm (admixture components within *japonica* and *indica*), GJ-trp (Southeast Asian tropical), GJ-sbtrp (Southeast Asian subtropical), GJ-tmp (primarily East Asian temperate), XI-1A (East Asia), XI-1B (modern varieties of diverse origins), XI-2 (South Asia), XI-3 (Southeast Asia), and XI-adm groups. The NIP and R498 genomes were selected as the representative *japonica* and *indica* reference genomes for the whole-genome comparison with LM8.

### 2.2 Detection and annotation of SNPs

The SNP databases for the 180 cultivated varieties are available online (SNP-Seek; http://snp-seek.irri.org). Information for 10 straw hull (SH) and 10 black hull awned (BHA) US weedy rice lines and 3 Chinese weedy rice lines was downloaded from the National Center for Biotechnology Information (NCBI) database (SRR4334499). The NIP, R498, and Arabidopsis genome data at the chromosome level were obtained from online databases (www.rice.uga.edu/, www.mbkbase.org/R498/, and www.ncbi.nlm.nih.gov/data-hub/genome/GCF_000001735.4/, respectively). Details regarding the LM8 genome are available in the NCBI BioProject database (accession number PRJNA754271).

Variant SNPs and insertions/deletions (InDels) were detected using the GATK (4.2.2.0) software, which was followed by (1) identification of active regions; (2) assembly of plausible haplotypes; (3) estimation of the per read likelihoods using the PairHMM algorithm; and (4) determination of the sample genotype. First, the GATK-HaplotypeCaller tool (versions 4.2.2.0) was used to analyze the bam files to obtain the sample GVCFs, after GATK-GenotypeGVCFs were used to genotype the variants with CombineGVCFs. Second, raw variants generated from the calling steps were filtered to identify the high-quality variants using the following parameters of GATK VariantFiltration: QD <2.0, MQ <40.0, FS >60.0, MQRankSum <−12.5, ReadPosRankSum <−8.0, and SOR >4.0. After combining the 3K-SNP data, the 5.12 Mb raw variants were filtered again (mis <10% and maf >5%) to generate the 4.03 Mb variant files (**Table S1**).

### 2.3 Population structure and phylogenetic analyses

On the basis of the whole-genome SNPs among the 201 *Oryza* species included in this study, a neighbor-joining phylogenetic tree was constructed using the Treebest (versions 1.9.2) software, with 1,000 bootstrap replicates. As previously described (Lee et al., 2014), a typical method to construct trees has been: 1) calculating p-distance from all SNP data between two samples, 2) making the p-distance matrix for all samples, 3) constructing and drawing the phylogenetic tree image. The principal component analysis (PCA) of the SNPs was completed using the GCTA software to cluster the principal components into different subsets according to the differences in the individual genome SNPs. The population structure was analyzed using the ADMIXTURE software. Low cross-entropy values reflected high-quality runs. Independent runs were performed for each simulated *K* value (from 2 to 10). The *K* value for which the cross-entropy curve reflected a sensible model was chosen (i.e., based on the likelihood value).

### 2.4 Genome comparison and analysis

Two comparisons were completed (LM8 *versus* NIP and LM8 *versus* R498) using the MUMmer (4.0.0rc1) software with the following parameters: nucmer -l 50 -c 100 -mum; delta-filter -i 90 -l 100 -1. According to the results of the collinearity analysis, presence-absence variations (PAVs) and structural variations (SVs) were identified using the SVUM and SYRI programs, respectively. In the final sequence variation files, variant sequences longer than 50 kb were retained, whereas variant sequences in the gap region were eliminated during the original variation test. Each SV was calculated for 1 Mb windows to generate the final Circos results.

### 2.5 *WRKY* and *NB-LRR* gene family analysis

To identify the *WRKY* and *NB-LRR* genes, the WRKY and TIR, CC, NBS, or LRR amino acid motifs were searched. The *WRKY* and *NLR* sequences in the LM8, NIP, and Arabidopsis genomes were obtained from database (accession number PRJNA754271 in the NCBI BioProject, www.rice.uga.edu, and www.ncbi.nlm.nih.gov/data-hub/genome/GCF_000001735.4/, respectively). The Molecular Evolutionary Genetics Analysis (MEGA; v11) program was used to construct a maximum-likelihood gene family phylogenetic tree, with 1,000 bootstrap replicates. Next, the *WRKY* and *NB-LRR* genes were mapped to 12 chromosomes using MapChart (2.3).

## 3 Results

### 3.1 Subpopulation classification of LM8 in *Oryza* plants

**Figure 1:**
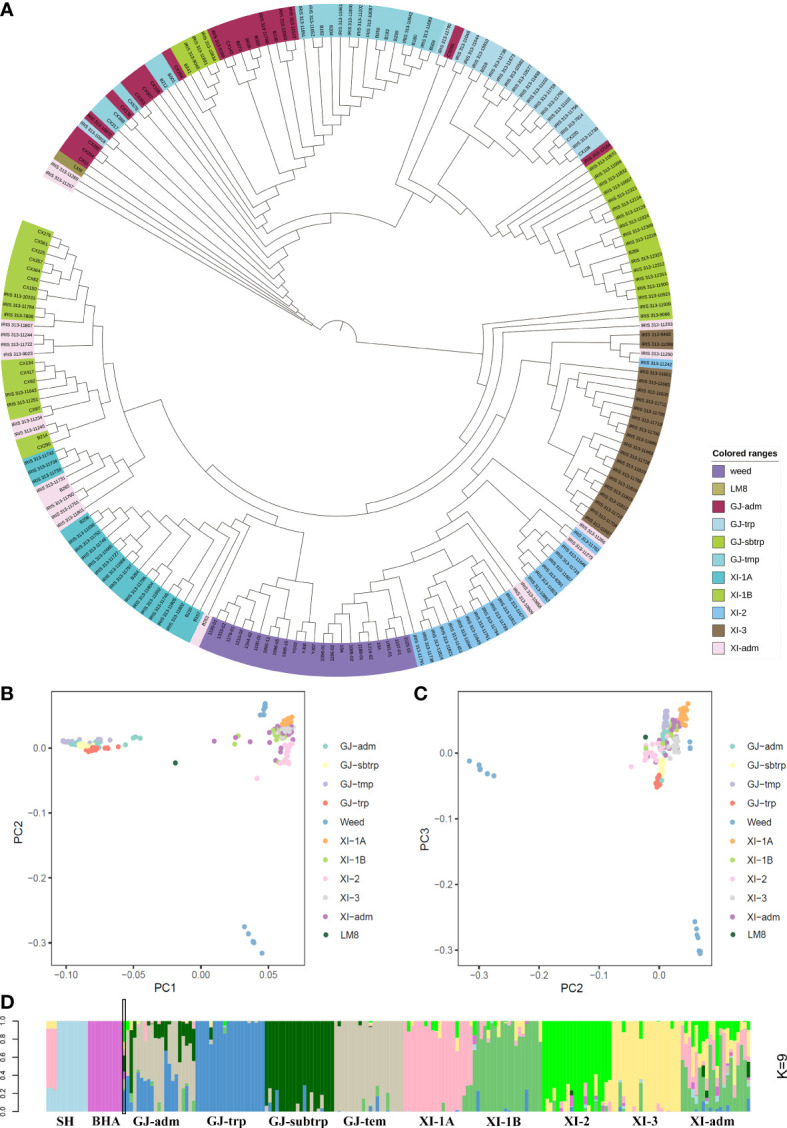
Subpopulation classification of LM8. LM8 was classified and its population structure was determined *via* an analysis of genome-wide SNPs in 203 cultivated and weedy rice lines. **(A)** Maximum-likelihood phylogenetic tree. **(B–C)** Principal component analysis. **(D)** Genetic proportions calculated using STRUCTURE ADMIXTURE (*K* = 9). GJ and XI represent the *O. sativa* groups *japonica* and *indica*, respectively. As described by Wang et al. (2018), GJ-adm and XI-adm represent admixture components within *japonica* and *indica*. Additionally, GJ-trp, GJ-sbtrp, GJ-tmp, XI-1A, XI-1B, XI-2, and XI-3 represent Southeast Asian tropical, Southeast Asian subtropical, primarily East Asian temperate, East Asia, modern varieties of diverse origins, South Asia, and Southeast Asia, respectively.

In 2021, Li et al. published the LM8 high-quality genome sequence and revealed LM8 is a *japonica*-type weedy rice line. To further classify LM8 within the *japonica* group, its SNPs as well as those in other weedy rice lines and accessions from the 3K database selected on the basis of the 3K classifications were downloaded to construct a neighbor-joining phylogenetic tree. The *indica* group (XI) served as the control. The phylogenetic tree constructed according to pairwise Nei’s genetic distances showed that the 10 subpopulations could be clustered into two groups (*japonica* and *indica*) (**Figure 1A**). Additionally, LM8 was located in the ‘adm’ branch (**Figure 1A**), implying that it was admixed (i.e., between *japonica* and *indica*). Moreover, LM8 was also classified in the *japonica* group. To verify the classification of LM8 in GJ-adm, a PCA was performed using the genome-wide SNPs in domesticated and weedy rice varieties (**Figures 1B, C**, and **S1**). The first three principal components (PCs), which explained 36.68% of the variance (**Table S2**), corresponded to genetic evolutionary factors as follows: PC1 separated the *japonica* (GJ-adm, GJ-trp, GJ-sbtrp, and GJ-tmp) and *indica* (XI-1A, XI-1B, XI-2, XI-3, XI-adm, SH, and BHA) subgroups (**Figures 1B, C**), whereas PC2 and PC3 distinguished between the weedy rice lines. Consistent with this finding, the weedy rice lines SH and BHA formed two clusters. The centralized subpopulation classification suggested the population clustering according to 3K was accurate. In terms of LM8, PC1 revealed the obvious differences between LM8 and the cultivated (*japonica* and *indica* groups) or weedy rice varieties (**Figure 1B**).

Next, the STRUCTURE program and an increasing subpopulation (*K*) value (2 to 10) were used to assign individuals to population structures and to explain the PC1-based clustering of LM8. The final population subgroups were determined according to (1) the likelihood value of these models and (2) the previously reported classification of accessions on the basis of the 3K database. When *K* was set to 2, the likelihood value was highest (0.570) and most of the rice accessions were clearly divided into the *indica* and *japonica* groups (**Figure S3**). Additionally, 50.43% and 49.57% suggested the “misplaced” LM8 was derived from a *japonica* and *indica* mixture (**Table S3**). Of the nine runs for *K* = 9, the run with the lowest likelihood value (0.462) was optimal for grouping samples and was selected for assigning the posterior membership coefficients to each accession (**Figure S3**). These rice accessions were grouped into the following 11 subpopulations: SH, BHA, GJ-adm, GJ-trp, GJ-sbtrp, GJ-tmp, XI-1A, XI-1B, XI-2, XI-3, and XI-adm (**Figure 1D**). In addition, the *indica*/*japonica* admixture of LM8 was easily identified and was in accordance with the phylogenetic relationships, with 22.98% from XI-2, 22.86% from GJ-trp, 17.76% from GJ- sbtrp, 9.86% from GJ-tmp, 8.63% from XI-3, 8.44% from BHA, 6.59% from XI-1A, 2.36% from XI-1B, and 0.52% from SH (**Table S3**). Nearly 10% of the genome contained genetic remnants of weedy rice, but in terms of geographical locations, LM8 tended to be similar to the tropical or subtropical accessions. These results suggested that the LM8 admixed genetic background was derived mostly from *indica*/*japonica* hybridizations rather than from weedy rice lines. Furthermore, LM8 is a *japonica*-type weedy rice line.

### 3.2 Whole-genome comparisons with NIP and R498

**Figure 2:**
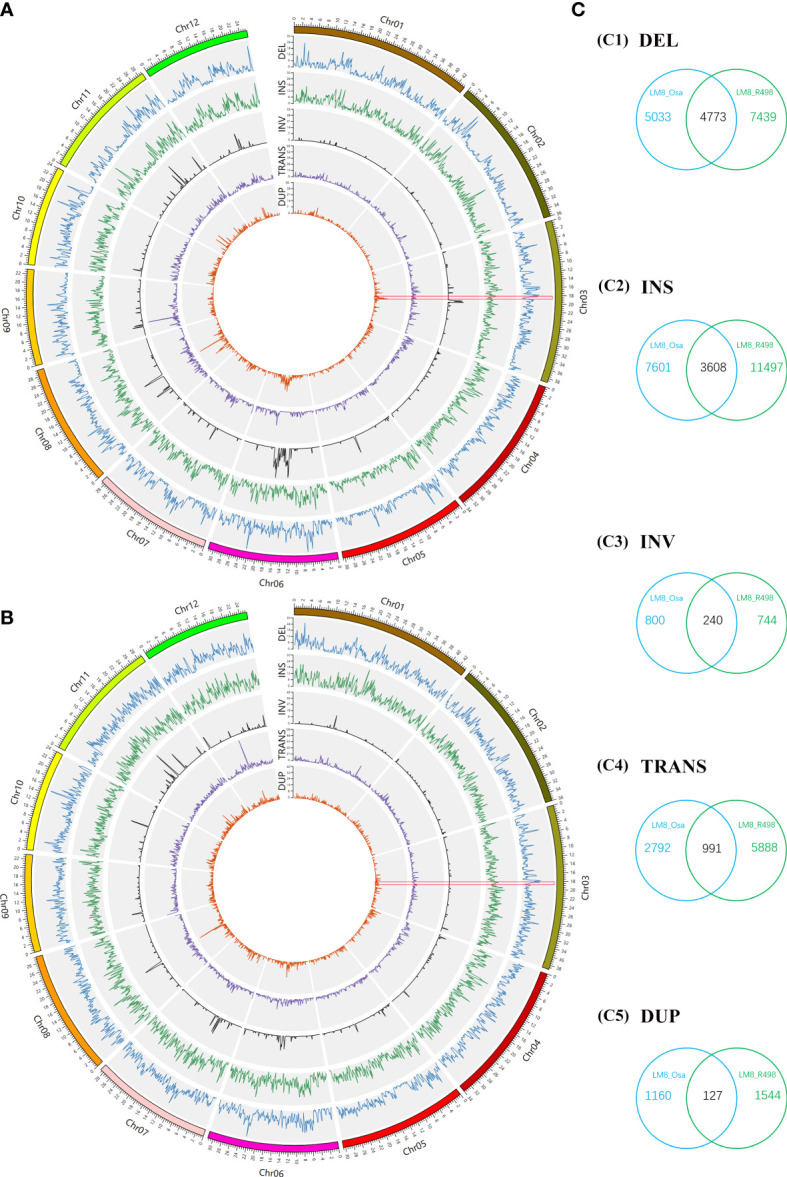
Whole-genome comparisons between LM8 and NIP and between LM8 and R498. **(A)** Circular diagram of the variation distribution and density on 12 chromosomes in LM8 revealed by the LM8 *vs* NIP comparison. **(B)** Circular diagram of the variation distribution and density on 12 chromosomes in LM8 revealed by the LM8 *vs* R498 comparison. **(C)** Venn diagram analysis of the number of variations. DEL, deletion; INS, insertion; INV inversion; TRANS, translocation; DUP, duplication.

Considering a considerable proportion of its admixture components was from XI and GJ, LM8 may be a novel rice resource with a complementary gene pool. To further explore genome-wide diversity, the PAVs and SVs in the genomes of NIP (a typical *japonica* variety), R498 (a typical *indica* variety), and LM8 were detected and examined. Next, the deletion (DELs), insertion (INSs), inversion (INVs), translocations (TRANS) and duplication (DUPs) were identified between LM8 and NIP (as reference) and between LM8 and R498 (as reference; **Figure 2**). No matter comparing to NIP or R498, DELs and INSs were distributed relatively evenly across 12 chromosomes in the LM8 genome (**Figures 2A, B**). In contrast, INVs, TRANS and DUPs were unevenly distributed across 12 chromosomes in LM8, with many of them detected on chromosomes 2, 5, 6, 7, 8, 10, 11, and 12 (**Figures 2A, B**). Comparisons to NIP, the higher variation density of INVs were detected in the position of Chromosome 3 (20000001-21000000), Chromosome 5 (16000001-17000000), Chromosome 6 (12000001-19000000), Chromosome 8 (6000001-8000000 and 12000001-13000000), Chromosome 11 (18000001-19000000) and Chromosome 12 (14000001-15000000; **Figure 2A**, **Table S4**). Besides, there was a sharp peak in the 5000001-6000000 region of chromosome 8 and 78 variation density of TRANSs (**Figure 2A**, **Table S4**). Also, the obvious variation density peak of DUPs were detected in the position of Chromosome 3 (20000001-21000000), Chromosome 5 (15000001-16000000), Chromosome 6 (12000001-20000000), Chromosome 7 (11000001-14000000), Chromosome 8 (5000001-15000000), Chromosome 11 (9000001-10000000), Chromosome 12 (14000001-18000000; **Figure 2A**, **Table S4**). Differently, the maximum value of TRANSs located the 12000001-13000000 position on Chromosome 12 in the comparison of LM8 and R498 group (**Figure 2B**, **Table S5**). And comparisons to R498, the distinct peak value of INVs and DUPs were detected in the 14000001-16000000, 9000001-12000000, 12000001-14000000, 14000001-15000000, 11000001-15000000 region of chromosome 6, 7, 8, 10, 11, respectively (**Figure 2B**, **Table S5**). According to the result of the above description, there were significant correlation between INVs and DUPs of distribution in both LM8 *versus* NIP and LM8 *versus* R498 comparisons.

As expected, there were fewer SVs between LM8 and NIP than between LM8 and R498. The LM8 *vs* NIP comparison revealed 27,125 SVs, of which 9,806 were DELs, 11,209 were INSs, 1,040 were INVs, 3,783 were TRANSs, and 1,287 were DUPs in LM8 (**Figures 2A, C**). Among the 36,760 SVs detected by the LM8 *vs* R498 comparison, 12,121 were DELs, 15,105 were INSs, 984 were INVs, 6,879 were TRANSs, and 1,671 were DUPs in LM8 (**Figures 2B, C**). We defined the SVs exclusive to NIP or R498 as specific SVs, whereas the SVs in both NIP and R498 were designated as common SVs. Of the 63,885 SVs revealed by the two comparisons, 9,739 were common SVs (15.24%), including 4,773 DELs, 3,608 INSs, 240 INVs, 991 TRANSs, and 127 DUPs in LM8. In contrast, 27.21% (17,386/63,885) and 42.29% (27,021/63,885) of the SVs were specific to the LM8 *vs* NIP and LM8 *vs* R498 comparisons, respectively.

### 3.3 WRKY transcription factor families

**Figure 3:**
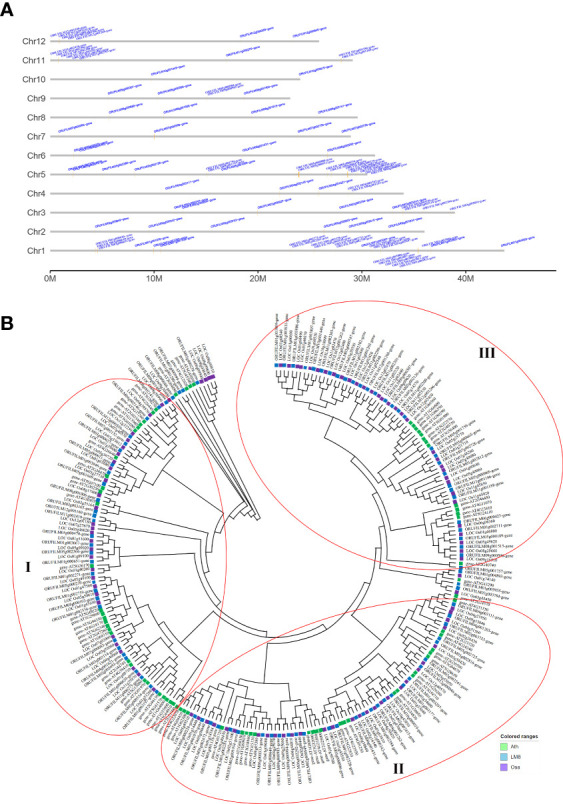
*WRKY* gene family analysis. **(A)** Distribution of *WRKY* genes across 12 chromosomes in the LM8 genome. **(B)** Phylogenetic tree containing *WRKY* genes from Arabidopsis, Nipponbare, and LM8. Ath, *Arabidopsis thaliana*; Osa, *Oryza sativa*. Nipponbare was selected as a typical cultivated rice variety, whereas Ath and Osa served as controls. The conserved WRKY structural domains were used to classify the *WRKY* genes, which are indicated by three red circles.

The WRKY TFs have core functions affecting plant hormone crosstalk and processes influencing plant growth and development (e.g., stress responses). It is unclear whether the abundant SVs in LM8 affect *WRKY* gene responses to various biotic and abiotic stresses under natural conditions. To identify *WRKY* genes and determine their chromosomal distribution, 96 *WRKY* genes in the LM8 and NIP genomes and 73 *WRKY* genes in the Arabidopsis genome were analyzed (**Figure S4**). Details regarding these genes, such as their gene IDs and WRKY domains and positions, are listed in **Table S6**. In LM8, *WRKY* genes were detected on 12 chromosomes, some of which were included in four clusters comprising at least four genes (**Figure 3A**). These clusters were distinct from those in Arabidopsis (**Figure S5**), with cluster 1 on chromosome 1 (35,398,161-35,468,751), cluster 2 on chromosome 5 (28,617,903-28,933,603), cluster 3 on chromosome 11 (756,297-803,420), and cluster 4 on chromosome 12 (854,961-904,182; **Table S6** and **Figure 3A**). Interestingly, 15 of the 18 *WRKY* genes in the four clusters belonged to group III. More specifically, the comparison with Arabidopsis indicated 13 of these 15 genes were clustered in subgroup IIIb (**Figure 3B**). These 13 *WRKY* genes were identified as follows: *ORUFILM01g003806*, *ORUFILM01g003807*, *ORUFILM01g003809*, *ORUFILM01g003811*, *ORUFILM11g002299*, *ORUFILM11g002300*, *ORUFILM11g002301*, *ORUFILM11g002302*, *ORUFILM11g002303*, *ORUFILM12g001262*, *ORUFILM12g001263*, *ORUFILM12g001266*, and *ORUFILM12g001268*.

The maximum-likelihood phylogenetic tree constructed using the *WRKY* gene sequences in LM8, NIP, and Arabidopsis and MEGA (v11) (**Figure 3B**) divided the genes into subgroups Ia, Ib, IIa, IIb, IIc, IId, IIIa, IIIb, and IV, which was in accordance with previously reported classifications (Ross et al., 2007). The group IV WRKYs lack a complete zinc-finger motif (Eulgem et al., 2000). An earlier study indicated japonica contains relatively few group IV WRKY TFs (Ross et al., 2007). In another study, Xie et al. (2005) proposed that group IV genes may be incorrectly annotated because of genome sequencing errors or they might be pseudogenes that lack biological functions. Thus, the three main groups of WRKY family genes (i.e., I, II, and III) are indicated by three red circles in **Figure 3B**. The phylogenetic analysis suggested group I diverged into two clades (subgroups Ia and Ib), whereas group II diverged into four clades, with subgroups IIa and IIb clustered in one clade and subgroups IIc and IId in another (**Figure 3B**). Group III was the largest, with subgroup IIIa and IIIb in two clades (**Figure 3B**). One of the subgroup IIIb members in LM8 was related to a gene in NIP (**Figure 3B**). In the phylogenetic tree, the following three genes were clustered with Arabidopsis genes and not NIP genes: *ORUFILM02g002693* (chromosome 2: 26,334,166-26,346,714), *ORUFILM05g002725* (chromosome 5: 1,733,309-1,734,147), and *ORUFILM05g001757* (chromosome 5: 15,083,202-15,087,922) (**Figure 3B**). Moreover, they were specifically detected in the LM8 genome and not in the NIP genome (**Figure 3A**). According to the constructed tree, *ORUFILM02g002693* is most closely related to *AT2G44745*, whereas *ORUFILM05g002725* and *ORUFILM05g001757* are most closely related to *AT2G23320* and *AT2G40740*, respectively (**Figure 3B**).

### 3.4 *NBS* gene families

**Figure 4:**
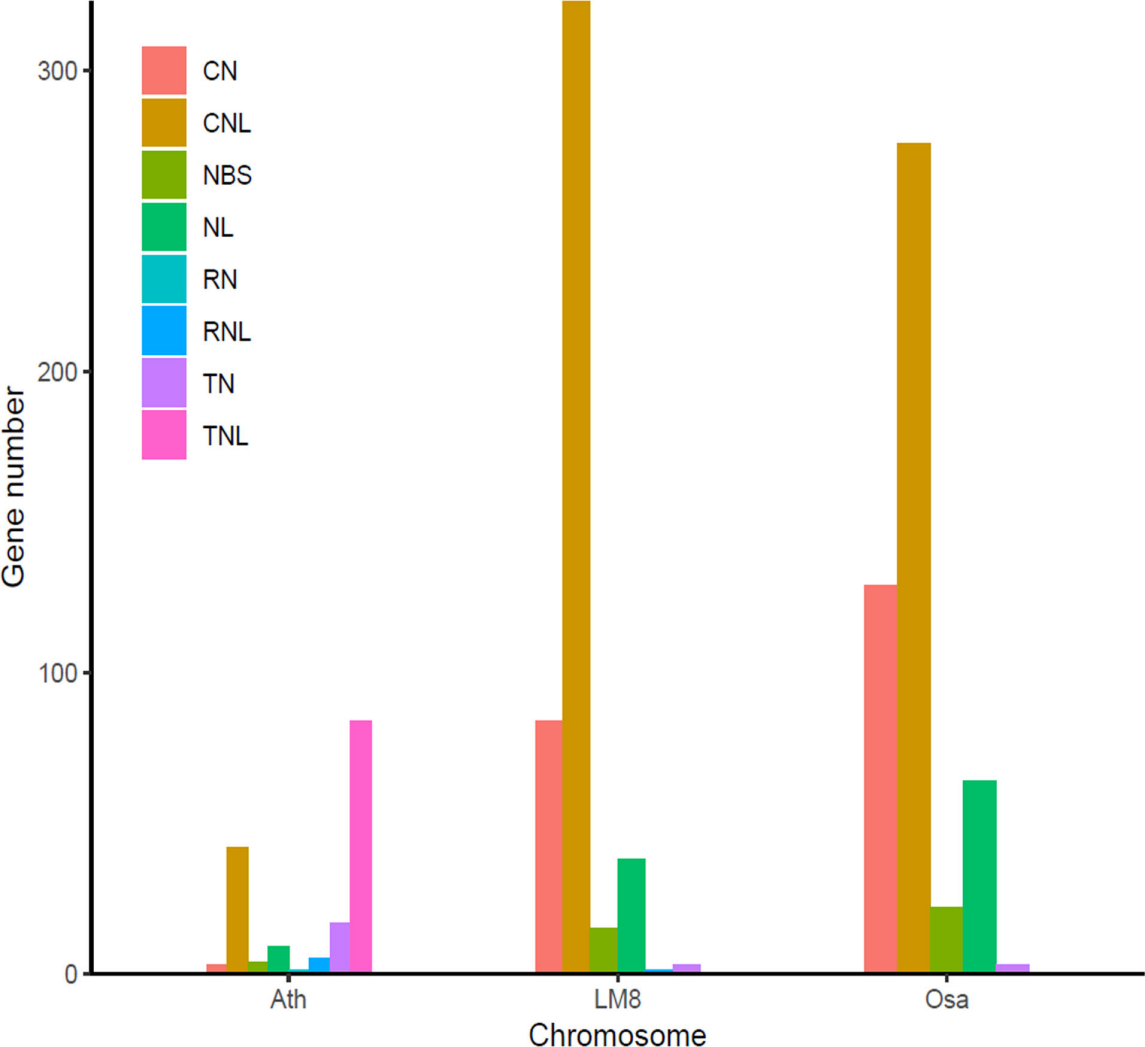
Number of different types of *NBS* genes in the LM8 genome. Ath, *Arabidopsis thaliana*; Osa, *Oryza sativa*. Nipponbare was selected as a typical cultivated rice variety, whereas Ath and Osa served as controls.

**Figure 5:**
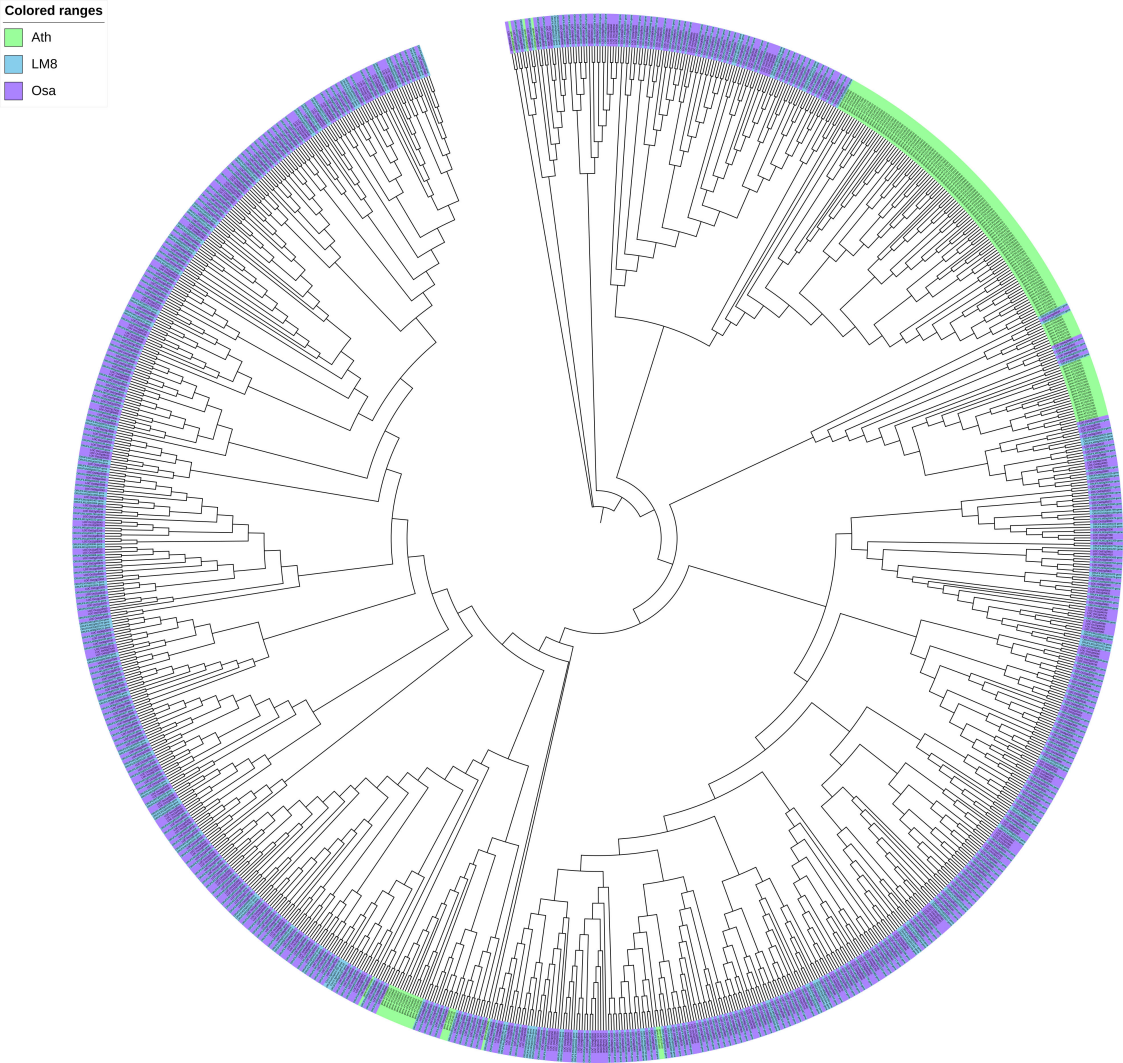
Phylogenetic analysis of *WRKY* genes in Arabidopsis, Nipponbare, and LM8. Ath, *Arabidopsis thaliana*; Osa, *Oryza sativa*. Nipponbare was selected as a typical cultivated rice variety, whereas Ath and Osa served as controls.

To clarify the pathogen resistance associated with sequence variants that may be applicable for breeding, the NBS-encoding genes in LM8, NIP, and Arabidopsis were downloaded and analyzed. The NIP genome had the most *NBS* genes (496), followed by the genomes of LM8 (464) and Arabidopsis (165) (**Table S7** and **Figure S6**). The *NBS* genes in Arabidopsis were detected on 5 chromosomes, while the *NBS* genes in LM8 and NIP were on 12 chromosomes. In Arabidopsis, the *NBS* genes were mainly designated as CNL and TNL types (Mondragón-Palomino et al., 2002), according to Xiang et al. (2017), which followed by CN, NBS, NL, RN, RNL, and TN types (**Figure 4**). With the exception of one RNL-encoding gene (*ORUFILM12g000772*) in LM8, the *NBS* genes in the LM8 and NIP genomes were mainly CN, CNL, NBS, NL, and TN types (**Figure 4**). These results are consistent with the findings of an earlier study, in which CN- and CNL-encoding genes were identified in *Oryza* species (i.e., non-TNLs) (Zhou et al., 2004). In the LM8 and NIP genomes, there were three TN-encoding genes that lacked the LRR domain (**Figure 4**). Compared with the NIP genome, the LM8 genome had fewer CN-encoding genes, but more CNL-encoding genes (**Figure 4**). To elucidate the evolutionary relationships among the predicted *NBS* genes, a phylogenetic tree were constructed based on the fact the diversity of the protein domain was generally consistent with that of the NBS region (**Figure 5**). The tree revealed a lack of specific clustering, with the *NBS* genes divided into almost 200 groups. One of the clades included most of the CN-encoding genes in LM8 and NIP and the TNL-encoding genes in Arabidopsis, implying they may share a common origin and may be functionally similar. Furthermore, the phylogenetic tree indicated the *NBS* genes in Arabidopsis and rice (LM8 and NIP) formed distinct clusters, unlike the *WRKY* genes (**Figures 3**, **5**).

## 4 Discussion

*Oryza sativa* has been divided into two major groups (*indica* and *japonica*). Advances in biotechnology-based research have clarified the differences among these two groups and other varietal types at the isozyme, DNA, and genome levels. The diverse 3K-RG accessions of Asian rice cultivars were classified into the XI-1A, XI-1B, XI-2, XI-3, XI-adm, GJ-adm, GJ-trp, GJ-sbtrp, and GJ-tmp subpopulations following a neighbor-joining method-based phylogenetic analysis of genomic SNPs. The results of the current study are consistent with previously reported findings regarding *japonica* rice (Wang et al., 2018), while also demonstrating that LM8 is closely related to GJ-adm subpopulations and may have originated in tropical or subtropical regions. Moreover, consistent with a previous report (Li et al., 2017), the weedy rice lines SH and BHA were included in the indica group and they were closely related to the XI-1A and XI-2 subpopulations, respectively (**Figure 1A**). These results directly supported the findings of earlier research on rice evolution and further indirectly confirmed the accuracy of our result that LM8 belongs to GJ-adm. Accordingly, LM8 may be a source of heat resistance genes that can be exploited by breeders. As expected, the LM8 genetic background was mostly derived from cultivated rice varieties and was generally not altered by selective breeding, thereby avoiding problems associated with distant hybridizations. In terms of the genome SVs, 27.21% and 42.29% of the specific SVs were revealed by the LM8 *vs* NIP and LM8 *vs* R498 comparisons, respectively. This may explain the detection of LM8 between *japonica* and *indica* during the PCA (PC1-3). Additionally, there were more specific SVs than common SVs, reflecting the clear difference between the evolution of LM8 and the evolution of NIP and R498. Besides, considering of yield-related traits in rice, the small grain is another representative characterize to LM8. As a results, the quantitative trait loci for grain length were localized to chromosome 3 (Li et al., 2021) in LM8. The detected sequence variations on chromosome 3 (18,000,001-19,000,000, **Table S4**, **S5**) suggested that the SVs of DELs or INSs may be associated with the production of short grains (**Figures 2A, B**). However, the almost identical positions of the INVs and DUPs will need to be further analyzed to uncover additional evolutionary relationships and functions.

Similar to other weedy rice lines, LM8 grows in rice fields. It has also survived because of stochastic introduction. Thus, the genetic resistance of LM8 and other weedy rice lines to biotic and abiotic stresses developed under complex environmental conditions (Guan et al., 2019; Jia and Gealy, 2018). Many plant *R* genes have been studied. For example, there are hundreds of representative *NBS* genes that confer resistance to a wide range of plant pathogens, including bacteria (Zhao et al., 2005), fungi (Li et al., 2018), nematodes (Esmenjaud et al., 1996), and viruses (Seo et al., 2006), as well as insects (Quisenberry and Clement, 2002). In addition, *NBS* genes belong to the most important class of resistance-related gene families and they encode proteins that recognize pathogen-secreted factors, thereby activating downstream signaling pathways leading to defense responses. To activate defense activities following the perception of pathogen signals, TFs (e.g., WRKYs) bind to plant-specific cis-regulatory elements and activate gene expression. Furthermore, diverse plant processes may be responsive to various WRKY TF family members during exposures to a variety of stresses (Viana et al., 2018). Hence, genome-wide analyses of *NBS* and *WRKY* genes may expand our understanding of the effects of the proteins encoded by these genes on stress resistance, but future investigations will need to further clarify the functions of these proteins under natural conditions and/or the complex network of associated responses.

Many WRKY TF-encoding genes have been identified in the genomes of *Oryza* relatives. For example, Ross et al. (2007) mapped non-redundant *WRKY* genes to individual chromosomes, which resulted in the identification of 102 and 98 *WRKY* genes in *indica* and *japonica*, respectively. A total of 89 *WRKY* genes in NIP (*japonica*) and 97 *WRKY* genes in the *Oryza nivara* genome have been identified and mapped to the corresponding chromosomes. Wu et al. (2005) detected 102 putative *WRKY* genes in the rice genome and compared them with Arabidopsis genes. Future research on *WRKY* genes may further elucidate their genetic diversity and contributions to stress resistance. In this study, 96 *WRKY* genes were identified in the weedy rice line LM8 and then clustered into nine groups. The distribution of the *WRKY* genes on 12 chromosomes resulted from a long de-domestication process and the evolution of *Oryza* species. Our findings suggest that the group III rice *WRKY* gene family expanded more quickly than the other gene families. This phenomenon likely contributed to the adaptive responses of rice to complex environmental conditions. By using an online resource (https://www.arabidopsis.org/), three genes in LM8 (*ORUFILM02g002693*, *ORUFILM05g002725*, and *ORUFILM05g001757*) were predicted to affect the flowering stage, embryo cotyledonary stage, and vascular leaf senescence stage.

The *NBS* genes encode proteins responsible for plant immune responses to pathogens. Most of these genes in rice were identified in earlier genome-wide re-sequencing analyses. In 2004, Zhou et al. identified 535 NB domain-encoding sequences in the NIP genome, but genes encoding TIR-NB-LRR proteins were not reported. The *NB-LRR* genes in the genomes of *O. rufipogon* Griff. wild rice lines Huaye 1 and Huaye 2 reportedly differ from the corresponding genes in two reference genomes. More than 108 of these genes were revealed by different comparisons (Huaye 1 *vs* 93-11, Huaye 2 *vs* 93-11, Huaye 1 *vs* NIP, and Huaye 2 *vs* NIP). Furthermore, these *NB-LRR* genes were mainly localized to chromosomes 2 and 11. In an earlier investigation, 2,688 *NB-LRR* genes served as queries for a BLAST search; these genes were anchored to 12 chromosomes in three rice cultivars and eight wild rice accessions (Rawal et al., 2018). In the present study, we determined that LM8 contains one RNL-encoding gene (*ORUFILM12g000772*). This gene includes sequences for the CC, NB-ARC, and LRR domains. The ORUFILM12g000772 protein sequence was downloaded and aligned with LOC_Os12g39620, At5g66900, At5g66910, and AtRWP8.1 (**Figure S7**). The analysis of the five alternatively spliced sequences of LOC_Os12g39620 (**Figure S7A**) suggested amino acids 1-251 may correspond to the Arabidopsis RPW8 domain (**Figure S7B, C**). As previously reported for Arabidopsis, the *RPW8* loci, including *RPW8.1* and *RPW8.2*, mediate the resistance to the oomycete and fungal pathogens responsible for downy mildew and powdery mildew, respectively (Xiao et al., 2001). Li et al. (2018) confirmed that the ectopic expression of *RPW8.1* leads to increased resistance to the blast fungus *Pyricularia oryzae* and the bacterial pathogen *Xanthomonas oryzae* pv. *oryzae*. The *ORUFILM12g000772* gene described herein will need to be functionally characterized to assess whether LM8 may be useful for breeding novel rice varieties with enhanced resistance to various diseases.

Although the weedy rice line LM8 is one of many Oryza resources, its diverse genetic background and GJ-adm subpopulation closely related to rice cultivars should be considered by rice breeders. The results of our whole-genome analysis of LM8 and the comparison with the NIP and R498 genomes revealed specific SVs, indicative of genes that may be useful for distinguishing between rice accessions. Perhaps not surprisingly, four unique resistance genes were identified, among which the RNL-encoding gene may confer broad resistance to multiple pathogens. Hence, global whole-genome comparisons and analyses may facilitate the classification of subpopulations and the identification of elite genes that may be exploited by rice breeding programs.

## Data availability statement

The datasets presented in this study can be found in online repositories. The names of the repository/repositories and accession number(s) can be found in the article/**Supplementary Material**.

## Author contributions

ZYH performed the research, analyzed the sequencing data, and wrote the first draft of the manuscript. QWY designed the study and edited the manuscript. WHQ and XMZ supervised the project and provided experimental advice. FL, YLC and LFZ prepared the supplementary materials. JFH, YYW, DJL, MX, WYF, YMN, WLG, SZW and ZRL retrieved and reviewed the relevant literature and downloaded publicly available data. All authors contributed to and approved the final manuscript.
