# Supplementary material for: Global whole-genome comparison and analysis to classify subpopulations and identify resistance genes in weedy rice relevant for improving crops
Source: Front Plant Sci. 2023 Jan 10;13:1089445. doi: 10.3389/fpls.2022.1089445 (PMC9872009; doi:10.3389/fpls.2022.1089445)
Supplement: Supplementary file 2 [file Image_1.pdf]

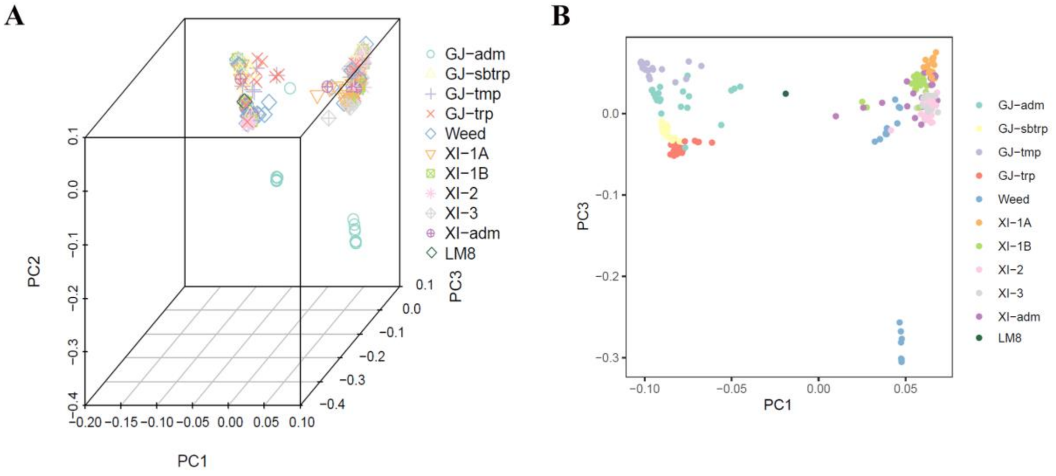

Figure S1 Principal component analysis of the genome-wide SNPs in cultivated and weedy rice

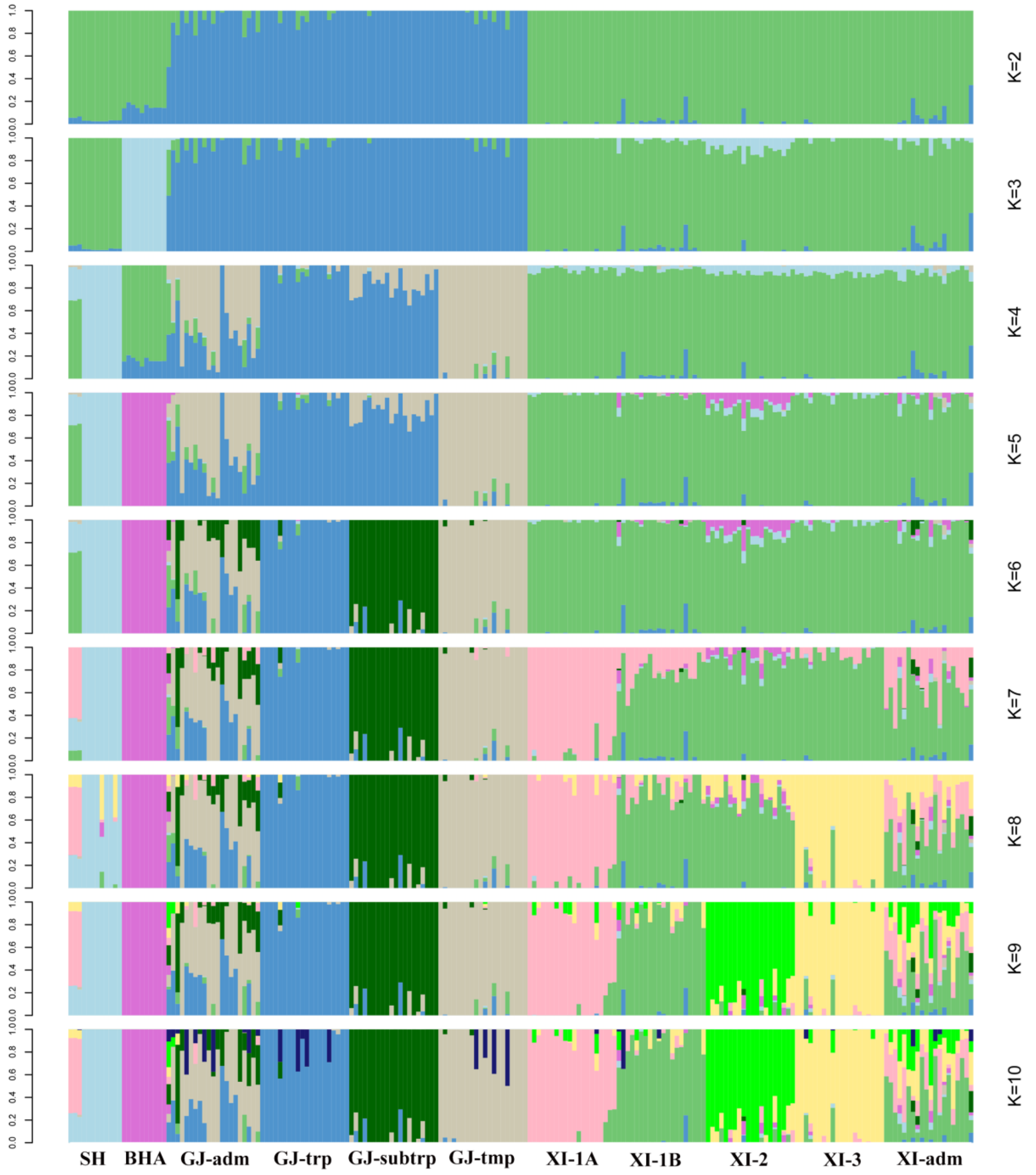

Figure S2 Genetic proportions calculated using STRUCTURE ADMIXTURE for *K* values ranging from 2 to 10

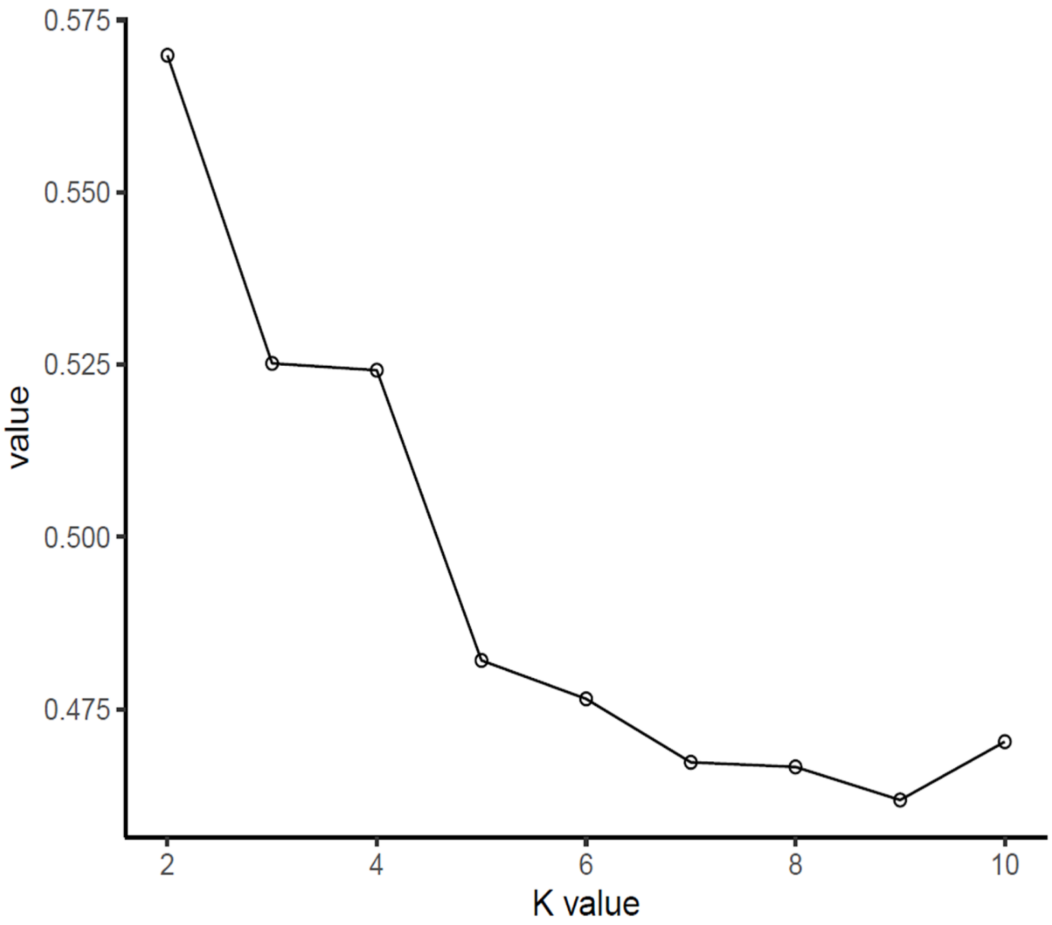

Figure S3 *K* value used for the STRUCTURE analysis

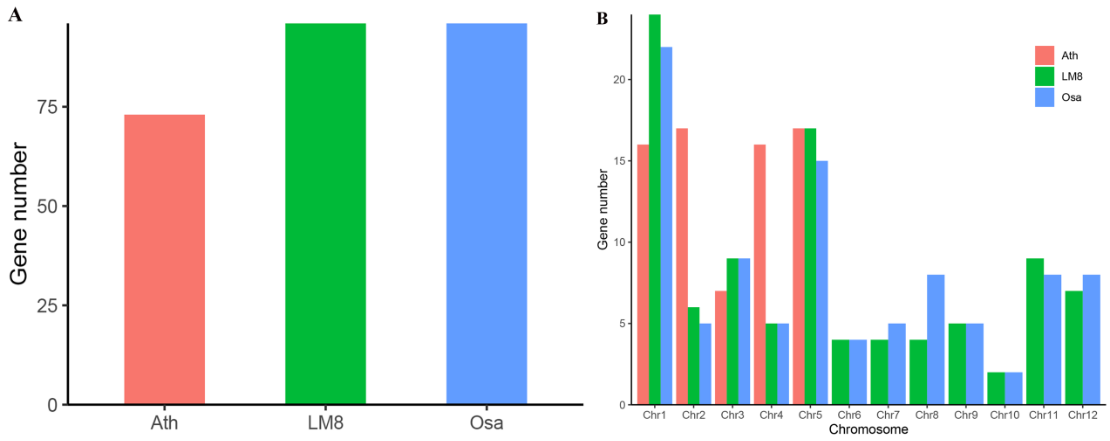

Figure S4 Number of *WRKY* genes in Arabidopsis, LM8, and NIP

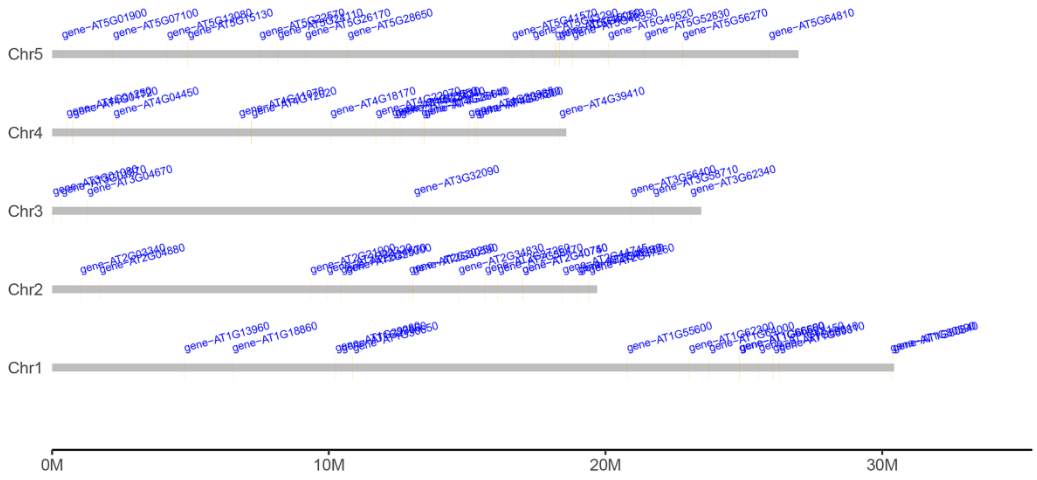

Figure S5 Distribution of *WRKY* genes among Arabidopsis chromosomes

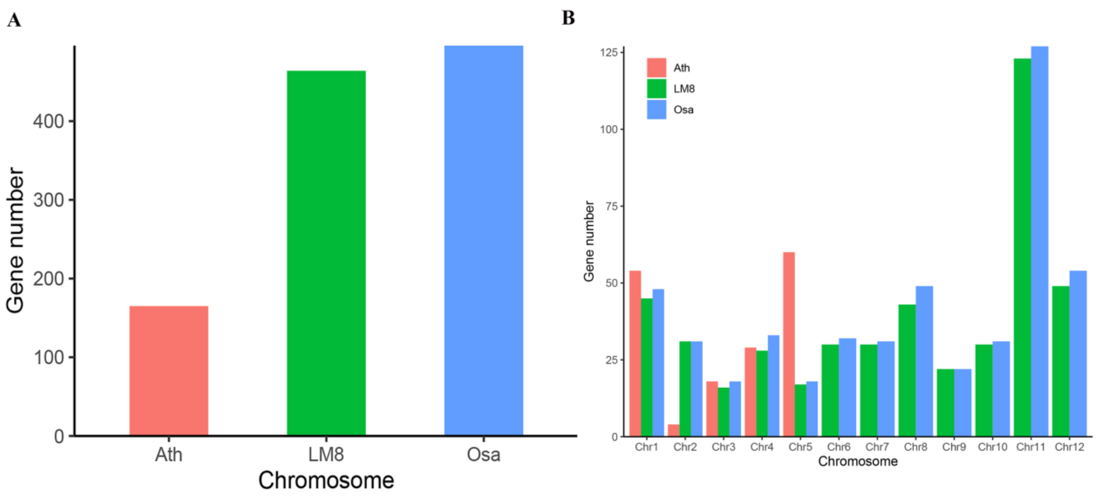

Figure S6 Number of *NBS* genes in Arabidopsis, LM8, and NIP

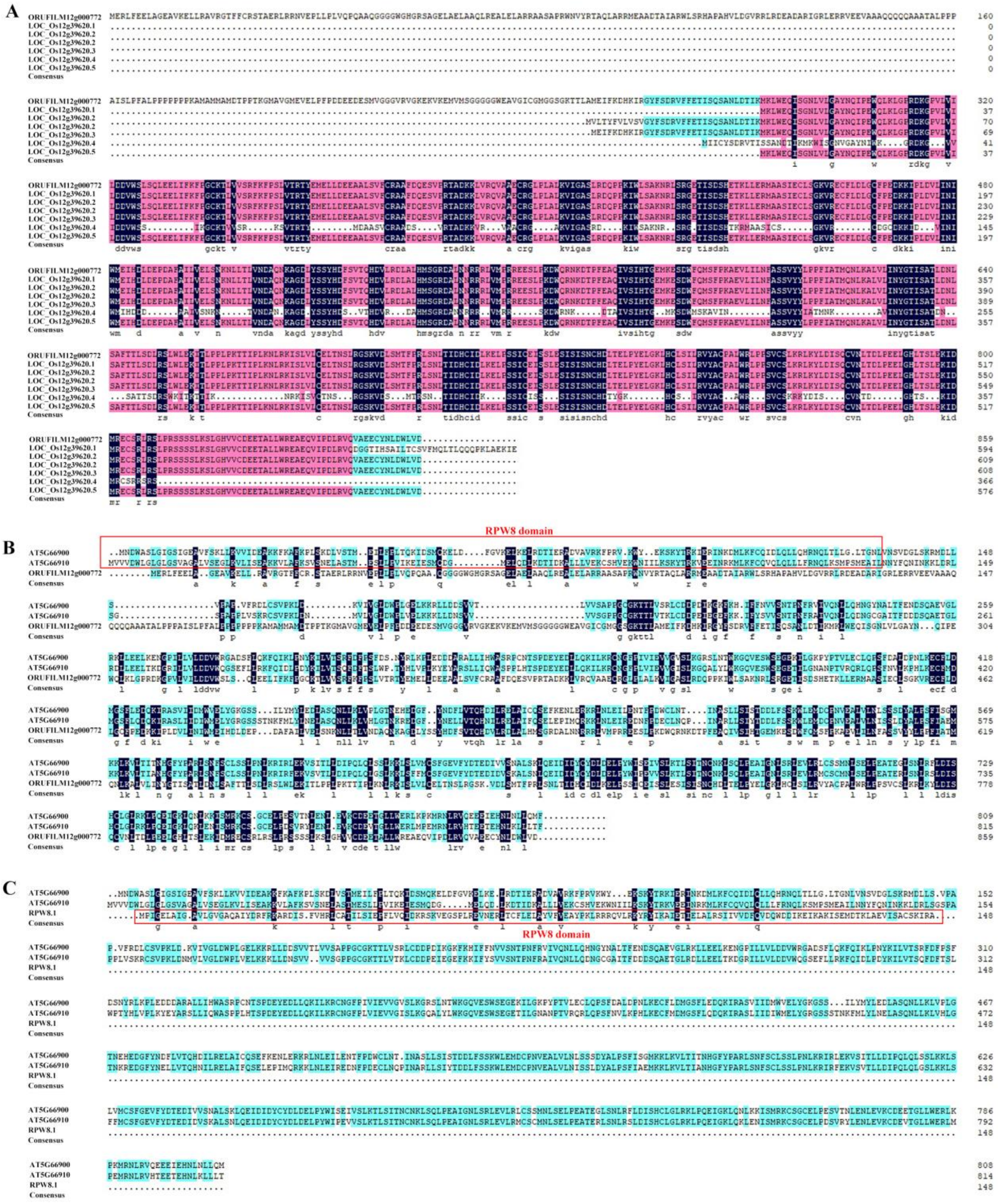

Figure S7 Alignment of ORUFILM12g000772, LOC_Os12g39620, At5g66900, At5g66910, and AtRWP8.1 protein sequences. The red box indicates the Arabidopsis RPW8 domain.
